# Expression of Semaphorin 4A and its potential role in rheumatoid arthritis

**DOI:** 10.1186/s13075-015-0734-y

**Published:** 2015-08-25

**Authors:** Lin Wang, Guanhua Song, Yabing Zheng, Weiwei Tan, Jihong Pan, Yu Zhao, Xiaotian Chang

**Affiliations:** Research Center for Medicinal Biotechnology, Key Laboratory for Rare & Uncommon Diseases of Shandong Province, Shandong Academy of Medicinal Sciences, Jinan, China; Institute of Basic Medicine, Shandong Academy of Medical Sciences, Jinan, China; Medical Research Center of Shandong Provincial Qianfoshan Hospital, Shandong University, Jingshi Road 16766, Jinan, Shandong 250014 People’s Republic of China; Department of Pathology, Shandong University Medical School, Jinan, China

## Abstract

**Introduction:**

Semaphorin 4A (Sema4A) plays critical roles in many physiological and pathological processes including neuronal development, angiogenesis, immune response regulation, autoimmunity, and infectious diseases. The present study aimed to investigate its expression and biological activity in rheumatoid arthritis (RA).

**Methods:**

RNA and protein were isolated from synovial tissues in RA and osteoarthritis (OA) patients. Treatment with recombinant human Sema4A (rhSema4A) or small interfering RNA (siRNA) was applied to examine its effect on the biological activity of synovial fibroblasts of RA (RASFs). Expression of Sema4A and NF-κB were measured by quantitative RT-PCR (qRT-PCR) and Western blot after lipopolysaccharide (LPS) stimulation. Chromatin immunoprecipitation (ChIP) and siRNA targeting p50 and p60 were applied to detect the regulation of Nuclear factor kappa (NF-κB) on Sema4A. Sema4A, interleukin 1β (IL-1β), interleukin 6 (IL-6), and tumor necrosis factor-α (TNF-α) secretion were measured by ELISA-based assays.

**Results:**

Increased levels of Sema4A were detected in the synovial tissue and fluid of patients with RA compared with those with OA. Furthermore, synovial fluid level of Sema4A correlated with Disease Activity Score (DAS) in RA. Treatment with rhSema4A promoted invasion of RASFs by upregulating the expression of Matrix metallopeptidase3 (MMP3), MMP9, alpha-smooth muscle actin(α-SMA), and Vimentin, and exacerbated inflammation by promoting the production of IL-6 in RASFs, as well as IL-1β and TNF-α in THP-1 cells. The induction of IL-6 and TNF-α by Sema4A was confirmed at the protein level in fluid samples from patients with RA. Knock-down experiments showed the participation of Plexin B1 towards rhSema4A in the induction of cytokines. In addition, LPS stimulation induced Sema4A expression in RASFs in an NF-κB-dependent manner, and rhSema4A treatment could also activate NF-κB signaling.

**Conclusions:**

These findings suggest an NF-κB-dependent modulation of Sema4A in the immune response. Further, increased expression of Sema4A is required to promote inflammation of RA.

**Electronic supplementary material:**

The online version of this article (doi:10.1186/s13075-015-0734-y) contains supplementary material, which is available to authorized users.

## Introduction

RA is characterized by chronic inflammation leading to progressive destruction of cartilage and bone [1]. Among the cells located in the inflamed joint, synovial fibroblasts are important players driving inflammation and bone erosion [1]. They are recognized as a source of cytokines such as IL-6 or receptor activator for nuclear factor-κ B ligand (RANKL), which activate immune response and osteoclastogenesis [2]. The study of molecules and mechanisms that regulate their biological activity could provide insight into the pathogenesis of rheumatoid arthritis (RA) and a basis for the development of new therapeutic strategies.

Semaphorins are a family of cell surface and soluble proteins originally identified as axon guidance factors that control the development of the central nervous system [3]. They are grouped into eight classes based on their structural domains, with classes 3–7 contributing the vertebrate semaphorins [4]. A previous study reported that decreased expression of semaphorin 3A is correlated with disease activity and histological features of RA [5]. Another family member, semaphoring 5A (Sema5A), contributes to the pathogenesis of RA through antigen-independent T cell and natural killer (NK) cell activation [6]. Further, patients with RA exhibit significantly elevated density of Sema3C-positive cells in synovial tissue when compared with patients with osteoarthritis (OA) or people without inflammation [7]. These findings underscore the relationship between semaphorins and RA.

In the immune system, Sema4A is preferentially expressed on dendritic cells (DC) and B cells [8, 9]. It has three known receptors: Tim-2, Plexin B1, and Plexin D1 [10, 11]. Sema4A activates a specialized and restricted genetic program in macrophages able to sustain angiogenesis and participates in their recruitment and activation in inflammatory injuries [12]. Recently, a Sema4A-neuropilin-1 (Sema4A-NRP1) axis was reported to maintain T regulatory (Treg) cell stability, highlighting this pathway as a potential therapeutic target that could limit Treg-cell-mediated tumor-induced tolerance without inducing autoimmunity [13]. However, to date, the exact expression amounts and function of Sema4A in RA, especially synovial fibroblasts of rheumatoid arthritis (RASFs), remain to be determined. Thus, in this study, we focus on the expression and biological activity of Sema4A in RA, which highlights its role in the pathogenesis of RA.

## Materials and methods

### Sample collection and cell culture

Synovial tissue and fluid samples were collected during knee joint replacement surgery from patients with RA (n = 12, 7 female, age 29 to 72 years old, mean 51 years) and patients with OA (n = 12, 6 female, age 39 to 77 years old, mean 62 years). All of the patients fulfilled the American College of Rheumatology (ACR) diagnosis criteria for RA and OA [14]. The RA patients had disease duration of 3–9 years and were classified as having erosive RA (Larsen class IV–V). These patients presented high levels of C-reactive protein (CRP) (32–298 mg/L, mean 73 mg/L), anti-cyclic citrullinated peptide (anti-CCP) (28–458 U/ml, mean 279.6 U/ml) and rheumatoid factor (RF) (38–316 U/ml, mean 183.2 U/ml). Before arthroplasty, the disease activity of each RA patient was evaluated using the 28-joint disease activity score based on CRP (DAS28-CRP) [15]. The degree of OA was graded by experienced orthopedic surgeons prior to surgery by x-ray classification, and the patients’ Kellgren and Lawrence grades ranged from 2–4. All of the patients provided written informed consent to participate in this study. The Ethics Committee of Shandong Provincial Qianfoshan Hospital approved this study. RASFs were isolated from synovial biopsy specimens of patients with RA as previously described [16] and cells between passages 4 and 7 were used for further study.

### Stimulation assays

RASFs were plated in 12- and 24-well plates (5 × 10^4^ and 3–5 × 10^5^ cells/well) in Dulbecco’s modified Eagle’s medium, and stimulated for the indicated time points with the following agents: lipopolysaccharide (LPS from *Escherichia coli* J5; SIGMA, St Louis, MO, USA), recombinant purified Sema4A (H00064218-P01, human, Abnova, Taiwan). The dilution buffer, PBS, was applied as the control to LPS or recombinant human semaphorin 4A (rhSema4A). Activation of signaling pathways was blocked using BAY 11–7082, an NF-κB inhibitor, WP1066, a Stat3 inhibitor, SU11274, a Met inhibitor or PD98059, a mitogen-activated protein kinase (MAPK) inhibitor (all from Calbiochem/EMD Millipore, Billerica, MA, USA).

### Small interfering RNA transfection in RASFs

RASFs (2 × 10^5^ cells in 100-mm-diameter dishes or 8.5 × 10^4^ cells in 6-well plates) were transiently transfected with Sema4A small interfering RNA (siRNA, 50 nM, SI00133560, SI04174107, QIAGEN, Hilden, Germany) or negative control (QIAGEN, SI03650318) by Hiperfect transfection reagent (QIAGEN) following the manufacturer’s instructions, and all experiments were performed 24–48 h after transfection. The specific siRNAs targeting p50/p65, Plexin B1, Plexin D1 and Tim-2 were designed and synthesized by GenePharma (Shanghai, China), and the most effective single siRNA was used for further experiments as follows: p65, sense strand: 5’-CCUCCUUUCAGGAGAUGAAUU-3’ and anti-sense strand: 5’-UUCAU CUCCUGAAAGGAGGUU-3’; p50, sense strand: 5’-GGCCUGAACAAAUGUUU CAUU-3’ and anti-sense strand: 5’-UGAAACAUUUGUUCAGGCCUU-3’; Plexin B1, sense strand: 5’-GGUUCUGGAUCAAUAUAAU-3’ and anti-sense strand: 5’-AUUAUAUUGAUCCAGAACC-3’; Plexin D1, sense strand: 5’- GGCCUCAAC UUGAUCUUCU-3’ and anti-sense strand: 5’- AGAAGAUCAAGUUGAGGCC-3’; Tim-2, sense strand: 5’- GCUGGGUUCAAGACUGUUA-3’ and anti-sense strand: 5’- UAACAGUCUUGAACCCAGC-3’. Non-specific negative control siRNAs were also designed and synthesized (sense strand: 5’-UUCUCCGAACGUGUCACG-3’ and anti-sense strand: 5’-ACGUGACACGUUCGGAGAATT-3’). The mock group was defined as that supplemented with the transfection reagent only.

### Cell invasion assay

The cell invasion assay was performed in transwell apparatus (Corning, Tewksbury, MA, USA). RASFs at a density of 3 × 10^4^ cells/well were grown to confluence and incubated with 100 nM of siRNA or rhSema4A in the upper compartment of the transwell apparatus for 48 h. After starvation, the lower compartment was filled with DMEM with 10 % FBS and the plates were continually incubated at 37 °C for 24 h. The upper surface of the insert was wiped with cotton swabs to remove non-invading cells, and the bottom surface of the insert was stained with Giemsa (Sigma, St Louis, MO, USA). The number of cells that invaded through the membrane was quantified in five random fields at × 100 magnification.

### ELISA

After various treatments, serum-free conditioned media samples were collected and centrifuged at 10,000 × g for 5 minutes to remove particulates. The supernatant was harvested and assayed for IL-1β, TNF-α, and IL-6 (R&D Systems, Minneapolis, MN, USA) by ELISA according to the manufacturer’s instructions. Sema4A within synovial fluid and culture media were determined as follows: sandwich ELISA was developed utilizing goat anti-Sema4A (C-46258, Santa Cruz, CA, USA) as capture antibody, rabbit anti-Sema4A (ab70178, Abcam, Cambridge, UK) as detection antibody and horseradish peroxidase-labeled goat anti-rabbit IgG antibody (Jackson, West Grove, PA, USA) together with 3,3′,5,5′-tetramethylbenzidin for color development. rhSema4A was tested at concentrations ranging from 1 to 1,000 ng/ml to generate a standard curve.

### Western blot analysis

Whole cell lysates were fractionated by 10 % SDS-PAGE, transferred to polyvinylidene difluoride (PVDF) membrane (Amersham Biosciences, Little Chalfont, UK) and probed with antibody to phospho-Stat3, Stat3; phospho-extracellular signal-regulated kinase (phosphor-ERK), ERK; phospho-MET, MET; phospho-NF-κB, NF-κB (Cell Signaling, Danvers, MA, USA), Sema4A (Abcam) or glyceraldehyde-3-phosphate dehydrogenase (GAPDH) (Santa Cruz). Bound antibodies were revealed with horseradish peroxidase-conjugated secondary antibodies (Santa Cruz) and the blot developed using an ECL Plus detection system (Thermo Scientific, Pittsburgh, PA, USA).

### RNA extraction and quantitative (qRT-PCR)

RNA extraction and qRT-PCR was performed as previously described [17]. The details of the primers are as followings: Sema4A: 5’-TAAAGTGAATGAAACCATTT GT-3’ (forward), 5’-GTCTGTGAAATGTTTTACAGTGT-3’ (reverse); GAPDH: 5’-C ACCATCTTCCAGGAGC-3’ (forward); 5’-AGTGGACTCCACGACGTA-3’ (reverse). GAPDH was used as internal loading control. Relative mRNA levels were measured using the 2-Δ cycle threshold (2-ΔCT) method. Three independent experiments were completed and each reaction was performed in triplicate.

### Chromatin immunoprecipitation assay (ChIP)

RASFs were untreated or treated with LPS for 24 h before the cells were fixed with 1 % formaldehyde in medium for 10 minutes at room temperature. The sonication conditions were optimized to determine generation of DNA fragments between 300 and 600 bp in length. Briefly, nuclear lysates were sheared with five to eight pulses using an Ultrasonic Processor (80 W) with tubes immersed in iced water. Each pulse cycle comprised 5 s sonication followed by 25 s cooling down. Chromatin was immunoprecipitated with IgG (Sigma) and anti-p65 (ab7970, Abcam). The association of p50 and p65 was measured by RT-PCR on immunoprecipitated chromatin by use of the following primers: 5’-TATACTGGCTGTGACA-3’ (forward) and 5’-TGACAGATCCTGGCTT-3’ (reverse).

### Statistical analyses

Statistical analysis of the data was performed using SPSS V.16 software (SPSS, Armonk, NY, USA). The *t* test was used to assess statistical differences between two groups. The correlation between Sema4A level and TNF-α, and IL-6 in serum from RA patients was calculated by Spearman’s correlation. *P* values <0.05 were considered significant. The data are presented as standard deviation.

## Results

### Expression of Sema4A in the synovial tissue and serum of patients with RA and its correlation with DAS28-CRP

**Fig. 1:**
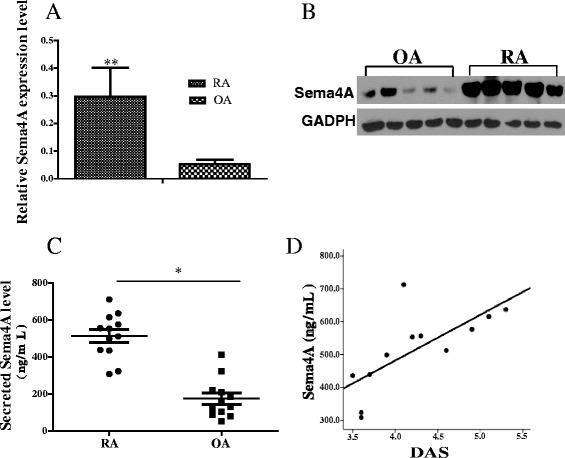
Increased expression of semaphorin 4A (*Sema4A*) in rheumatoid arthritis (*RA*). **a** Sema4A mRNA (relative to glyceraldehyde-3-phosphate dehydrogenase (GAPDH)) was detected by qRT-PCR and its expression increased in the synovial tissues in RA (n = 12) compared with those in osteoarthrits (*OA*) (n = 12). **b** Western blot analysis showed that Sema4A protein increased in the synovial tissues of RA (n = 12) compared with those of OA (n = 12) (ratio Sema4A/GAPDH RA 2.39 ± 0.15; OA 0.42 ± 0.12, *P* = 0.016 < 0.05). **c** The level of Sema4A was detected by ELISA in fluid from RA and OA patients. **d** Correlation between soluble Sema4A in fluid from RA patients (n = 12) and disease activity score in 28 joints (*DAS28*). *P <0.05 and **P <0.01 (statistically significant differences)

To study the association between Sema4A and chronic joint inflammation in RA, we first compared the expression profile of *Sema4A* mRNA in the synovial tissue of patients with RA and those with non-inflammatory OA. On qRT-PCR analysis there was a 5.56-fold higher level of Sema4A in synovial tissues of patients with RA than those with OA (Fig. 1a). Similarly, on western blot analysis the Sema4A protein level was also higher in the synovial tissues of patients with RA than those with OA (Fig. 1b). We next performed ELISA to determine the secreted level in a small cohort of patients with RA (n = 12) and OA (n = 12). A significantly increased level of Sema4A was detected in fluid from RA patients (514.3 ± 121.5 ng/mL) compared to that from OA patients (174.9 ± 105.8 ng/mL) (Fig. 1c). Then, we examined the correlation between secreted Sema4A and DAS28-CRP, the disease activity score for RA. Importantly, there was positive correlation between soluble Sema4A and DAS28-CRP (*r*^2^ = 0.504, *P* = 0.015; Fig. 1d). This suggests that the increased Sema4A expression might be involved in the disease activity of RA.

### Investigating the effect of Sema4A on the invasion of RASFs

**Fig. 2:**
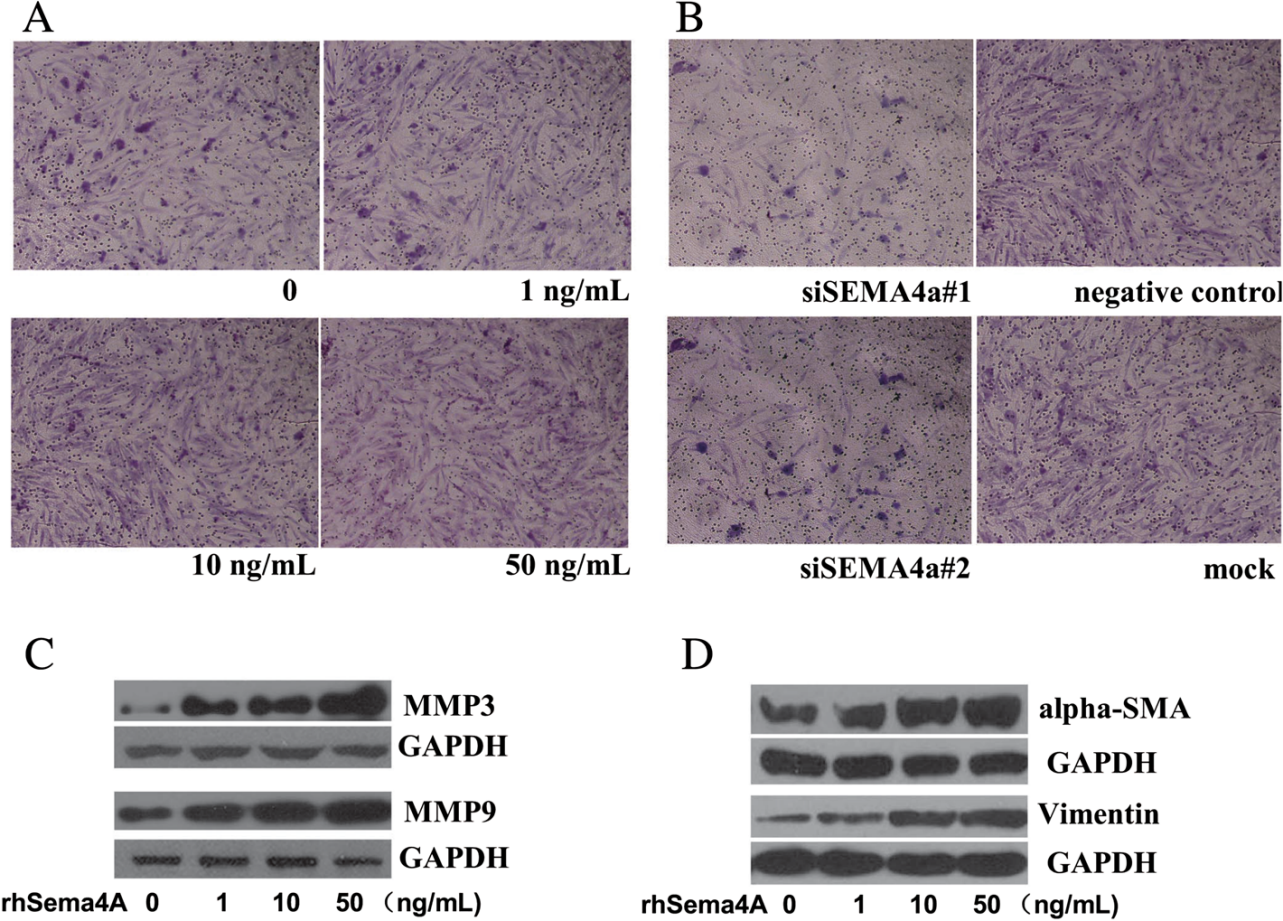
Semaphorin 4A (*Sema4A*) regulates the invasive phenotype of synovial fibroblasts of rheumatoid arthritis (RASFs). The invasive ability of RASFs as investigated by transwell apparatus after recombinant human semaphorin 4A (*rhSema4A*) (**a**) or silencing Sema4A (**b**) treatment, which were depicted in × 100 magnification. Western blot was applied to detect the expression of matrix metalloproteinase (*MMP*)3 and MMP9 (**c**), as well as alpha smooth actin (*α-SMA*) and vimentin (**d**) in RASFs after rhSema4A treatment at different concentrations. *GAPDH* glyceraldehyde-3-phosphate dehydrogenase

The effect of Sema4A on the biological activity of human RASFs was examined. RASFs were treated with various concentrations of rhSema4A in serum-containing medium for 24 h and the invasive viability was determined using the transwell chamber. As shown in Fig. 2a, the in vitro invasive ability increased when RASFs in the upper compartment were stimulated with rhSema4A at different concentrations for 24 h. In contrast, silencing Sema4A (100 nM) with siRNA showed that the invasion was blocked in comparison with the control groups (Fig. 2b). Matrix metalloproteinases (MMPs) degrade the extracellular matrix, promoting cell invasion and their upregulation has also been linked to the pathological process of RA [18]. To further support the pro-invasion ability of Sema4A, we then examined the effect of Sema4A on MMP expression. As shown in Fig. 2c, rhSema4A treatment induced MMP-3 and MMP-9 expression, which are important for cell invasion [18]. In addition, RASFs, undergoing myofibroblast or epithelial-mesenchymal transition (EMT)-like transition, would be more likely to invade [19, 20]. Accordingly, our data showed that rhSema4A treatment could also promote the above transition as demonstrated by the induction of the myofibroblast maker, α-smooth muscle actin (SMA) and the EMT marker, vimentin (Fig. 2d) [19, 20].

### Examining Sema4A expression following stimulation with LPS

**Fig. 3:**
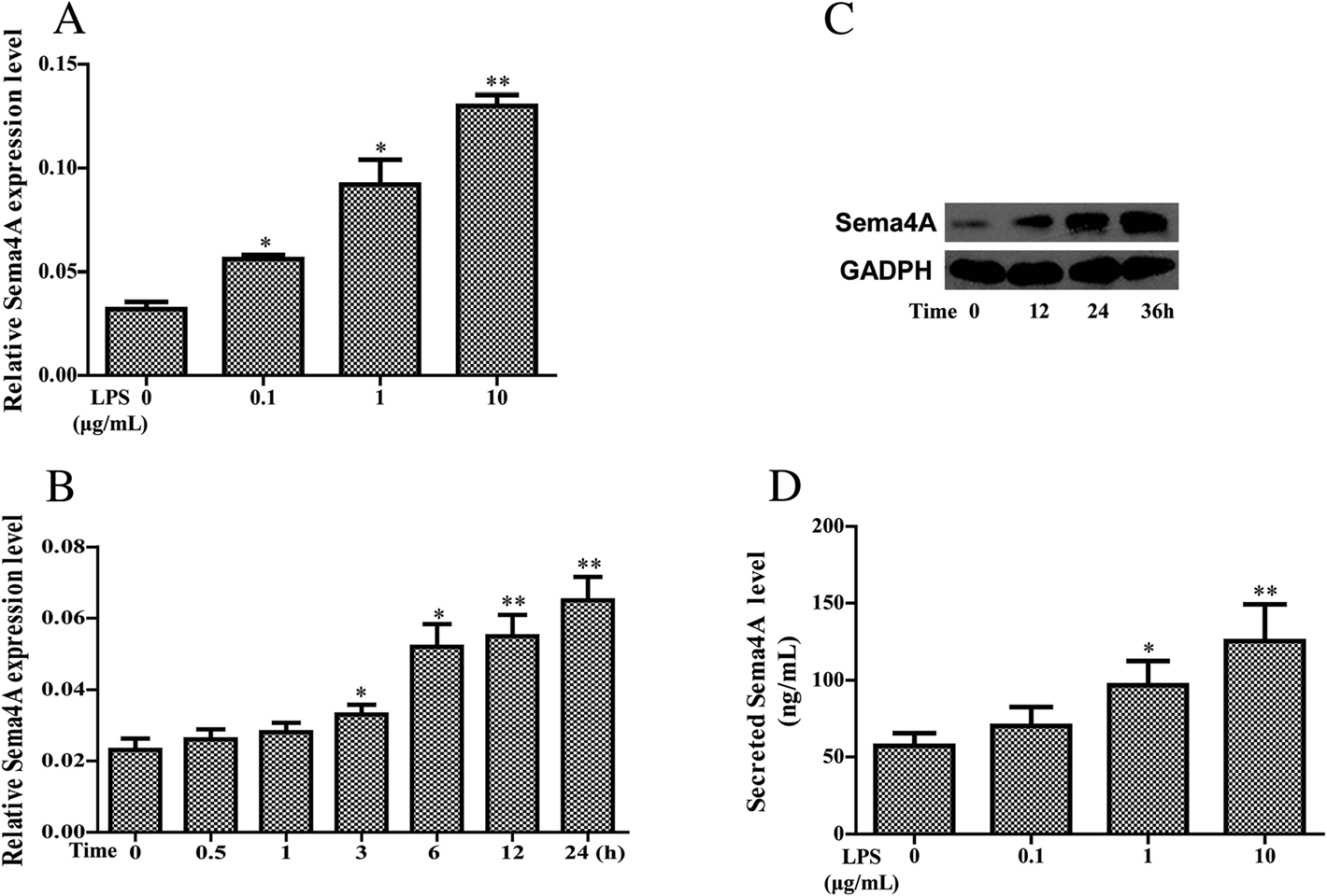
Effects of lipopolysaccharide (*LPS*) on the expression and secretion of semaphorin 4A (*Sema4A*). qRT-PCR was applied to detect the expression of Sema4A after stimulation by LPS at various concentrations (**a**) or periods (**b**) in synovial fibroblasts of rheumatoid arthritis RASFs, respectively. **c** Sema4A protein was assayed by western blot after LPS treatment in RASFs at indicated time points. **d** Secretion of Sema4A in the supernatant of RASFs was detected by ELISA after LPS stimulation. *P <0.05 and **P <0.01 (statistically significant differences) *GAPDH* glyceraldehyde-3-phosphate dehydrogenase

To analyze the influence of Sema4A in immune response, RASFs were treated with the toll-like receptor (TLR) ligand [21] LPS for 24 h. On qRT-PCR there was a modest induction of *Sema4A* mRNA expression after stimulation of RASFs with LPS at different concentrations (Fig. 3a). Time-course analysis showed that *Sema4A* expression increased after 3 h upon stimulation with LPS (1 μg/mL, Fig. 3b). Western blot analysis of LPS-stimulated RASFs (1 μg/mL) confirmed the induction of Sema4A protein after 12 h (Fig. 3c). Further, the secreted level of Sema4A in the supernatant of RASFs increased steadily after LPS stimulation (Fig. 3d). These findings prompted us to study the function of Sema4A during inflammation in detail.

### Regulation of Sema4A expression by NF-κB signaling

**Fig. 4:**
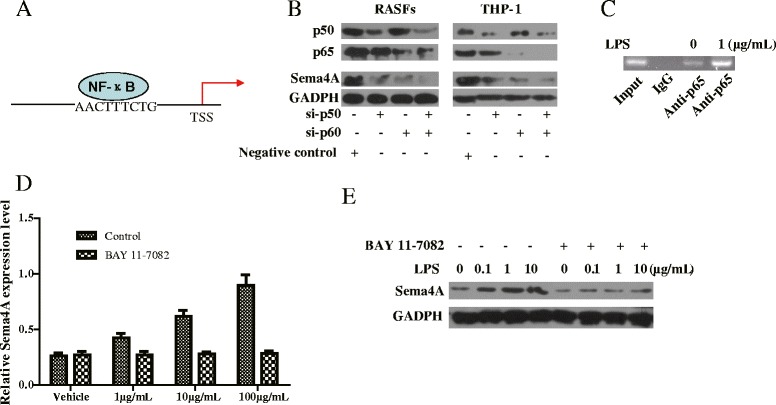
Regulation of semaphorin 4A (*Sema4A*) expression by NF-κB. **a** Schematic illustration as the binding of NF-κB to the promoter of Sema4A. **b** Synovial fibroblasts of rheumatoid arthritis (*RASFs*) and THP-1 cells were transfected with 50 nM of control, p50 or p65-siRNA, or p50 and p65 combined and incubated for the indicated time. Total extracts were separated by SDS-PAGE and western blot analysis was conducted for measurement of Sema4A protein levels. **c** Chromatin immunoprecipitation (ChIP) analysis showed that p65 was recruited to the Sema4A promoter after treatment with lipopolysaccharide (*LPS*) for 90 minutes. On ChIP analysis with control primers against distinct promoter regions there was no recruitment of p65. Input, chromatin input before immunoprecipitation; Anti-p65, immunoprecipitated chromatin with anti-p65 antibody; IgG, immunoprecipitated chromatin with control IgG. Effect of NF-κB inhibitor treatment on the LPS-induced Sema4A was detected by qRT-PCR (**d**) and western blot (**e**)

Using the position-weight matrices available in GeneCards (Genomics for SEMA4A Gene, Regulatory Elements for SEMA4A Gene) [22], an NF-κB binding site on the Sema4A promoter was identified (Fig. 4a). To further clarify the role of the NF-κB subunits p50 and p65 in the regulation of Sema4A [23], we used siRNA knockdown (both 100 nM) to silence p50 and p65 in RASFs. siRNA constructs effective at silencing p50 or p65 (Fig. 4b), individually and in combination, also inhibited Sema4A expression (Fig. 4b). We also confirmed the above results in THP-1 cells with p50 and p65 knocked down individually and in combination, resulting in suppressed Sema4A expression (Fig. 4b). As NF-κB could regulate Sema4A expression at the transcriptional level, we next investigated whether NF-κB could directly bind to its promoter region. The results of ChIP analysis showed that more p65 was bound to the Sema4A promoter after treatment (Fig. 4c). Importantly, blocking NF-κB signaling attenuated the LPS-induced expression of Sema4A at the transcriptional (Fig. 4d) and translational (Fig. 4e) levels, which demonstrates that the increased recruitment of NF-κB to the Sema4A promoter after LPS stimulation is indeed responsible for its induction in RASFs.

### Production of IL-6 by Sema4A in RASFs

**Fig. 5:**
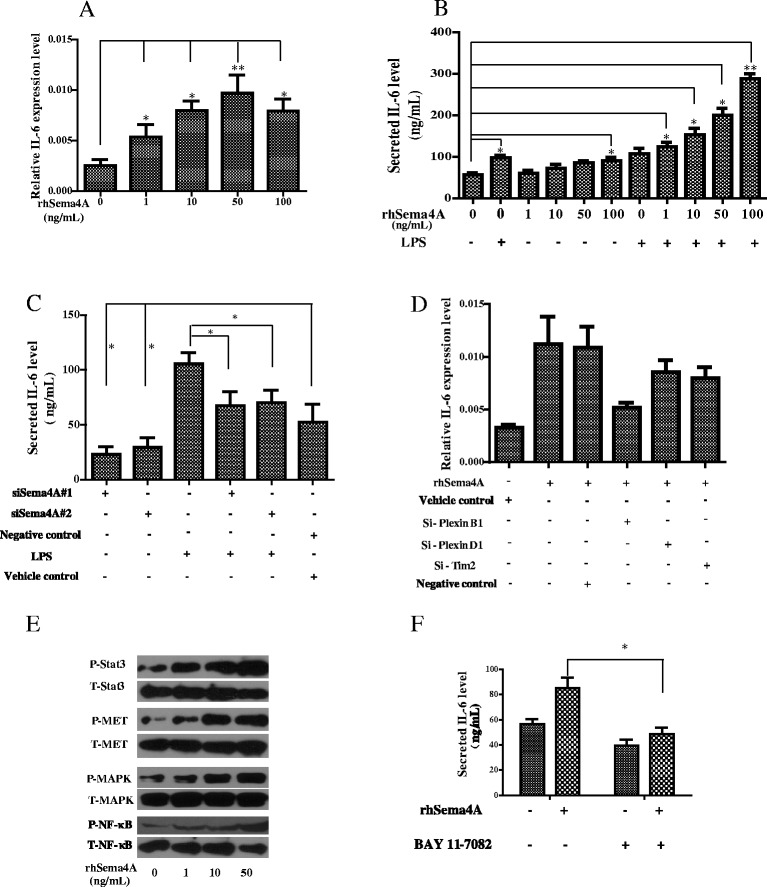
Semaphorin 4A (*Sema4A*) promotes IL-6 production in an NF-κB dependent way. **a** Synovial fibroblasts of rheumatoid arthritis (RASFs) were treated with recombinant human semaphorin 4A (*rhSema4A*) at different concentrations and the expression of IL-6 was assayed by qRT-PCR. **b** Effects of rhSema4A, with or without lipopolysaccharide (LPS) (1 μg/ml) stimulation, on the secretion of IL-6 in the supernatant of RASFs was detected by ELISA. **c** After silencing Sema4A expression in RASFs, the IL-6 secretion was also detected by ELISA. **d** The expression of IL-6 in RASFs was detected by qRT-PCR after silencing Plexin B1, Plexin D1 or Tim-2, respectively. **e** The phosphorlation of Stat3, mitogen-activated protein kinase (*MAPK*), Met and NF-κB signaling in RASFs was analyzed by western blotting after treatment with rhSema4A (10 ng/ml) for the indicated periods. **f** IL-6 secretion in the supernatant of RASFs was determined by ELISA after various treatments. *P <0.05 and **P <0.01 (statistically significant differences)

The production of the proinflammatory cytokine IL-6 was analyzed in rhSema4A-stimulated RASFs with or without LPS stimulation (1 μg/ml). Interestingly, rhSema4A treatment induced the expression (Fig. 5a) and secretion of IL-6 (Fig. 5b) in a dose-dependent manner. Notably, rhSema4A and LPS showed a duplicate effect on the induction of IL-6 in RASFs (Fig. 5b). Accordingly, when RASFs were transfected with control or Sema4A-specific siRNA for 48 h, there was significant reduction of both basal and LPS-stimulated expression of IL-6 after knocking down Sema4A (50 nM, Fig. 5c). The basal IL-6 production was reduced by 75 ± 7.2 % (*P* <0.05), whereas LPS-induced IL-6 levels were reduced by 34.7 ± 3.8 % (*P* <0.05) (Fig. 5c).

### Mediation of NF-κB during Sema4A functions

In further examining the pathological involvement of Sema4A, our qRT-PCR analysis showed that silencing of Plexin B1 attenuated the induction of IL-6 after rhSema4A treatment more significantly than silencing of other receptors (Fig. 5d). Next, we wondered whether Sema4A induced phosphorylation of signaling molecules in RASFs. On immunoblot rhSema4A treatment upregulated the phosphorylation levels of STAT3, MAPK, MET, and NF-κB in a dose-dependent manner (Fig. 5e). We next examined the effects of various inhibitors targeting major signaling pathways on Sema4A-induced IL-6 upregulation in RASFs. As a result, rhSema4A-induced (50 ng/ml) IL-6 production was significantly inhibited by addition of BAY 11–7082 (10 uM), an NF-κB inhibitor, but not by WP1066, a STAT3 inhibitor (Fig. 5f), SU11274, a MET inhibitor, or PD98059, an MAPK inhibitor for ERK (data not shown). These findings suggest that Sema4A induces IL-6 production in RASFs via the NF-κB pathway.

### Effect of Sema4A on the induction of TNF-α and IL-1β by LPS-activated THP-1 cells

**Fig. 6:**
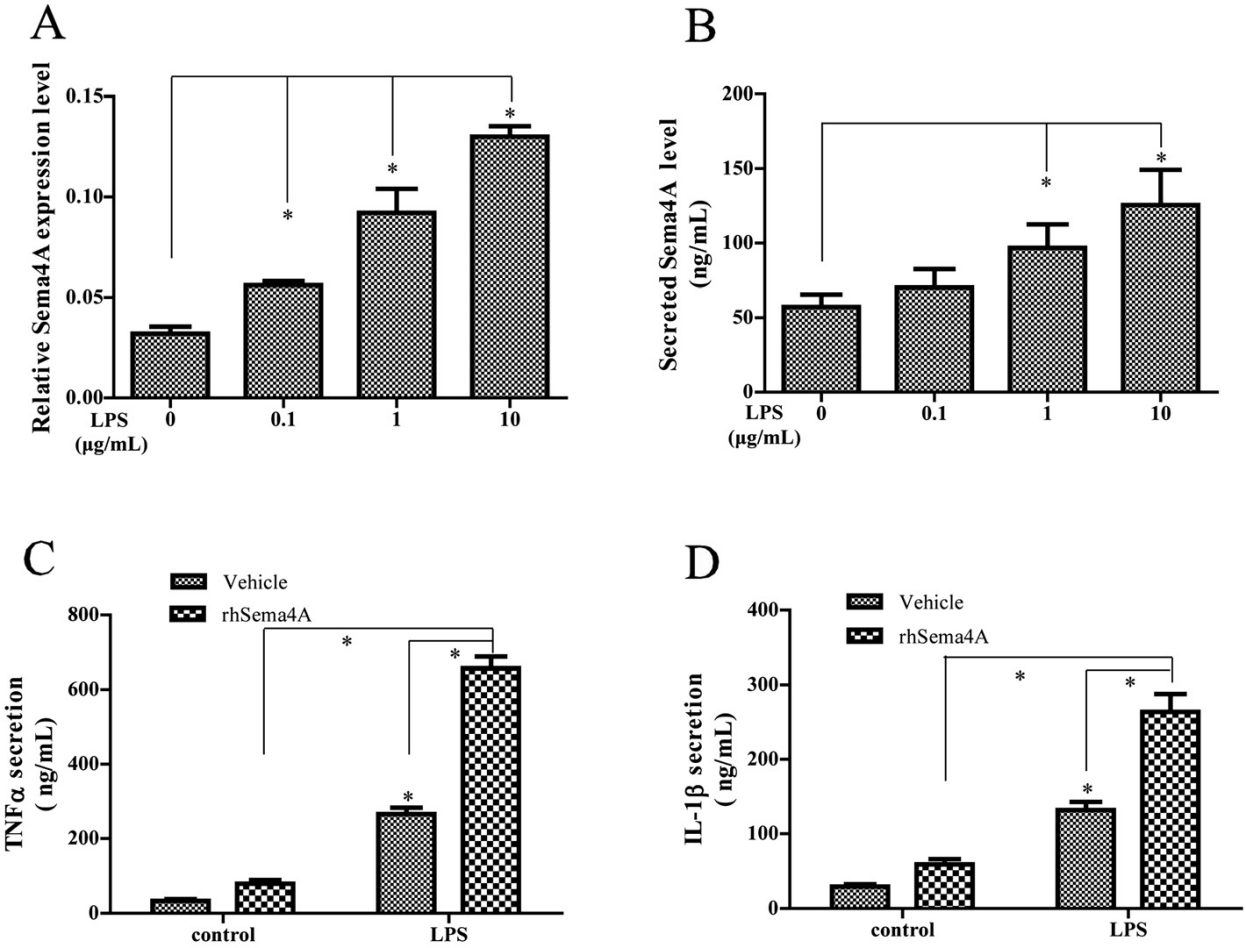
Semaphorin 4A (*Sema4A*) promotes TNF-α and IL-1β production in lipopolysaccharide (*LPS*)-activated THP-1. Expression (**a**) and secretion (**b**) of Sema4A were analyzed by qRT-PCR and ELISA in THP-1 after LPS stimulation. Secretion of TNF-α (**c**) and IL-1β (**d**) was determined by ELISA after recombinant human semaphorin 4A (*rhSema4A*), or in combination with LPS treatment. *P <0.05 (statistically significant differences)

As RASFs do not secrete either TNF-α or IL-1β [24], we next determined whether Sema4A overexpression could affect the production of these cytokines by macrophages, which also play an important role in RA. We thus treated the macrophage cell line THP-1 with LPS and performed qRT-PCR analysis of Sema4A. In these cells Sema4A expression was also strongly upregulated in a dose-dependent manner upon LPS treatment (Fig. 6a). We confirmed that as in RASFs, LPS treatment also induced Sema4A secretion in THP-1 cells (Fig. 6b). We next tested whether Sema4A affected release of TNF-α and IL-1β. rhSema4A treatment for 48 h induced TNF-α and IL-1β more so than cells treated with PBS vehicle alone (Fig. 6c, d). Next, we measured TNF-α and IL-1β production by THP-1 cells treated with Sema4A for 48 h and then activated with LPS (1 μg/ml) for 24 h. Compared with the control and consistent with the observations made in RASFs, we observed that Sema4A significantly promoted the release of these cytokines in response to LPS, compared with the control without LPS stimulation (Fig. 6c, d).

### Correlation of the serum levels of Sema4A with TNF-α and IL-6 in RA patients

**Fig. 7:**
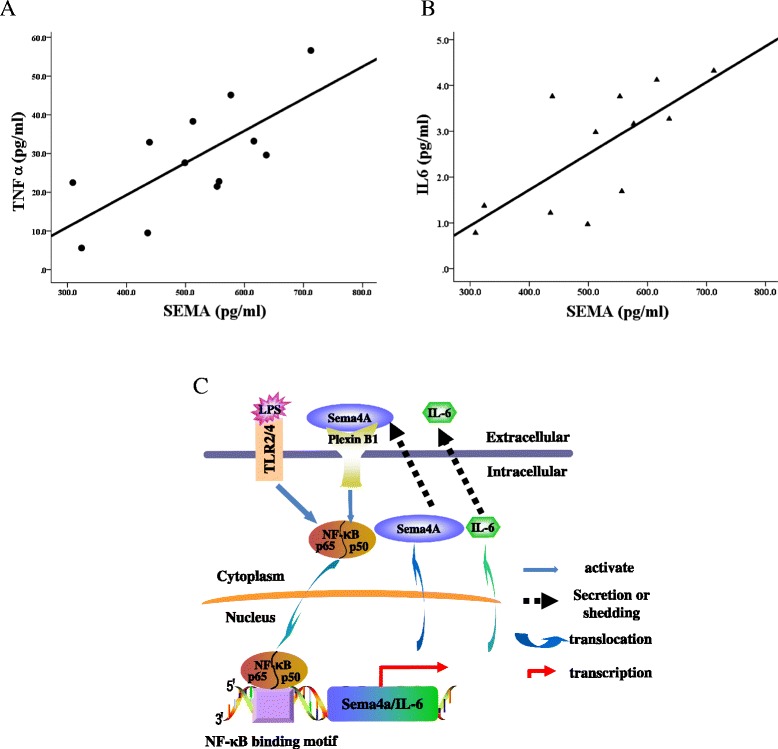
Correlation between serum semaphorin 4A (*Sema4A*) and cytokine levels in patients with rheumatoid arthritis. ELISA was performed to quantify the serum levels of Sema4A, IL-6, and TNF-α in RA patients. Correlation between serum Sema4A and the level of IL-6 (**a**), and TNF-α (**b**) assessed by Pearson correlation analysis. **c** A putative model illustrating the interactions of Sema4A/NF-κB in modulating cell phenotype through control of IL-6 production

Serum TNF-α and IL-6 levels were measured to better determine the roles of Sema4A in RA. Significant correlation was found between Sema4A and IL-6 levels (*r*^2^ = 0.723, *P* = 0.008, Fig. 7a) and between Sema4A and TNF-α levels (*r*^2^ = 0.711, *P* = 0.01, Fig. 7b). Sema4A levels were not significantly correlated with IL-1β (data not shown). These results further support the pro-inflammatory role of Sema4A in RA.

## Discussion

Previous studies showed that semaphorins such as Sema3A, Sema3C, and Sema5A are involved in the pathogenesis of RA [5–7]. Our results here demonstrated first that the expression levels of Sema4A are significantly higher in the synovial tissue and serum of patients with RA compared to OA; this finding is consistent with that of other semaphorins such as Sema5A. Second, we demonstrated that Sema4A is involved in the inflammatory response in RA. Bacterial and viral infections are associated with the occurrence and pathogenesis of flare reactions in RA [25] and the critical involvement of TLR activation by its ligand, LPS, in the initiation and persistence of inflammation in RA is well recognized [26]. Here, the induction of Sema4A expression by LPS, together with the expression of Sema4A in monocytes/macrophages [12], indicates its involvement in persistent synovial inflammation in RA. Further, Sema4A secretion levels also correlate with disease activity, further supporting its role in the progression of RA.

In high-inflammation synovial tissues, RASFs can undergo a process resembling EMT, a phenomenon found in early developmental processes, tissue repair, fibrosis, and carcinogenesis [19]. Additionally, RASFs exhibit a gene expression profile characteristic of myofibroblasts (e.g., α-SMA and vimentin) [20]. In contrast to other semaphorin studies focusing on immune cells such as T and B cells, our study evaluated the regulation of Sema4A on the biological activity of RASFs. Interestingly, our data showed that Sema4A can induce EMT by upregulating α-SMA and Vimentin expression in RASFs. Additionally, Sema4A promotes the production of MMPs (MMP3 and MMP9), which degrade the extracellular matrix (ECM) [18], thereby providing space for RASFs to invade. These findings suggest that Sema4A is a key effector disturbing the joint microenvironment and a driving force that directs proliferative synovium to destroy cartilage and bone in RA.

Previous studies showed that Sema4A-mediated functions are mediated via receptors such as TIM-2, Plexin B1, and Plexin D1 [10, 11]. Our study demonstrated that the induction of IL-6 by rhSema4A was significantly inhibited by silencing Plexin B1, and partially inhibited by silencing Plexin D1 and TIM-2. Therefore, during the exacerbation of RA, although the expression of Sema3C and Sema5A, together with Sema4A, increases in the synovial tissues and serum of patients with RA, these proteins may function in a cell-specific or condition-specific manner that offsets their likely functional redundancy.

IL-6, IL-1β, and TNF-α are all important cytokines that stimulate RASFs and inflammatory cells to aggravate synovial inflammation, resulting in joint destruction [27, 28]. Our study also confirmed that Sema4A can exacerbate the pathological progression by regulating the production of these cytokines in a synergistic way with LPS. Importantly, a significantly positive correlation was identified between Sema4A expression and the production of TNF-α and IL-6 in the serum of RA patients. These data show multiple levels of interactions between Sema4A and cytokines in vitro that occurs during inflammation in the presence of TLR ligands, and suggests this as a potential treatment target for RA. It has been reported that Sema4A can maintain the Treg cell stability in tumors [13]. The microenvironment of RA, however, is different to that of tumors, and the balance of Treg cells is dependent on the localized cytokine milieu [13]. Thus, the therapeutic effects of inhibiting Sema4A merits further study, which also may increase our understanding of the role of Tregs in RA.

To further determine the possible mechanism of action of Sema4A, we explored intracellular signal transduction pathways. Previous studies demonstrated that NF-kB could be activated through Plexin B1 in endothelial cells [29]. Here, the effect of Sema4A was linked to the intracellular activation of NF-κB, MAPK, and STAT3 pathways, all of which represent major signaling cascades involved in the activation of RASFs [30–32]. In particular, production of IL-6 is NF-κB dependent, which is essential for cytokine network triggering and amplifying that escort for the chronic inflammatory responses of RA, largely through regulating the expression of pro-inflammatory mediators such as TNF-α, IL-1β, and IL-6 [27, 28]. Interestingly, our data showed that Sema4A is a direct target of NF-κB, and blocking NF-κB signaling attenuates LPS-induced Sema4A expression and induction of IL-6 after rhSema4A treatment. Although the suppression of p50 or p65 did not completely eliminate the expression of Seme4A because of the silencing efficiency or some other regulatory factors towards Sema4A, these findings indicate that NF-κB is required for inflammation in RA, as mediated by Sema4A. Interestingly, rhSema4A treatment also induced Sema4A expression in RASFs (Additional file 1: Figure S1), which may be due to an additional effect caused by the activation of NF-κB signaling. Collectively, these Sema4A-mediated effects support the presence of a positive loop between components of innate immunity, such as stimuli, cytokines, monocytes/macrophages, and cells from the synovial stroma, such as RASFs, which may be critical to the exacerbation of inflammation in RA.

## Conclusion

In summary, we report the overexpression of Sema4A in RA synovial tissue and fluid. In Fig. 7c, we propose a model to illustrate how Sema4A/NF-κB promotes inflammation in RA through modulation of IL-6. Sema4A activates NF-κB signaling through PlexinB1, which in turn binds to the promoter of IL-6 and facilitates its transcription. Notably, this effect can be strengthened by LPS treatment.

Interestingly, Sema4A can also induce its own expression in an NF-κB-dependent manner. Although *in vivo* experiments are needed, Sema4A inhibition seems to have a dual therapeutic benefit, acting on two aspects of RA pathogenesis (synovial invasion and inflammatory response), reinforcing the potential utility of Sema4A inhibitors in RA.

## Additional file

Additional file 1: Figure S1. Recombinant human semaphorin 4A (*rhSema4A*) treatment induced its own expression in synovial fibroblasts of rheumatoid arthritis (RASFs). (JPEG 759 kb)

## Abbreviations

α-SMA: alpha-smooth muscle actin
anti-CCP: anti-cyclic citrullinated peptide
bp: base pairs
ChIP: chromatin immunoprecipitation assay
CRP: C-reactive protein
DAS: disease activity score
DC: dendritic cells
DMEM: Dulbecco’s modified Eagle’s medium
ELISA: enzyme-linked immunosorbent assay
EMT: epithelial-mesenchymal transition
ERK: extracellular signal-regulated kinase
FBS: fetal bovine serum
GAPDH: glyceraldehyde-3-phosphate dehydrogenase
IL-1β: Interleukin 1β
IL-6: Interleukin 6
LPS: lipopolysaccharide
MMP: matrix metallopeptidase
MAPK: mitogen-activated protein kinase
NF-κB: nuclear factor kappa B
NK: natural killer cell
NRP1: neuropilin-1
OA: osteoarthritis
PBS: phosphate-buffered saline
PVDF: polyvinylidene difluoride
RA: rheumatoid arthritis
RANKL: receptor activator for nuclear factor-κ B ligand
RASFs: synovial fibroblasts of rheumatoid arthritis
rhSema4A: recombinant human semaphorin 4A
SDS-PAGE: Sodium dodecyl sulphate polyacrylamide gel electrophoresis
Sema4A: semaphorin 4A
siRNA: small interfering RNA
TLR: toll-like receptor
TNF-α: tumor necrosis factor-α
Treg: T regulatory
