# Supplementary figures and images for: Expression of Semaphorin 4A and its potential role in rheumatoid arthritis
Source: Arthritis Res Ther. 2015 Aug 25;17(1):227. doi: 10.1186/s13075-015-0734-y (PMC4549119; doi:10.1186/s13075-015-0734-y)

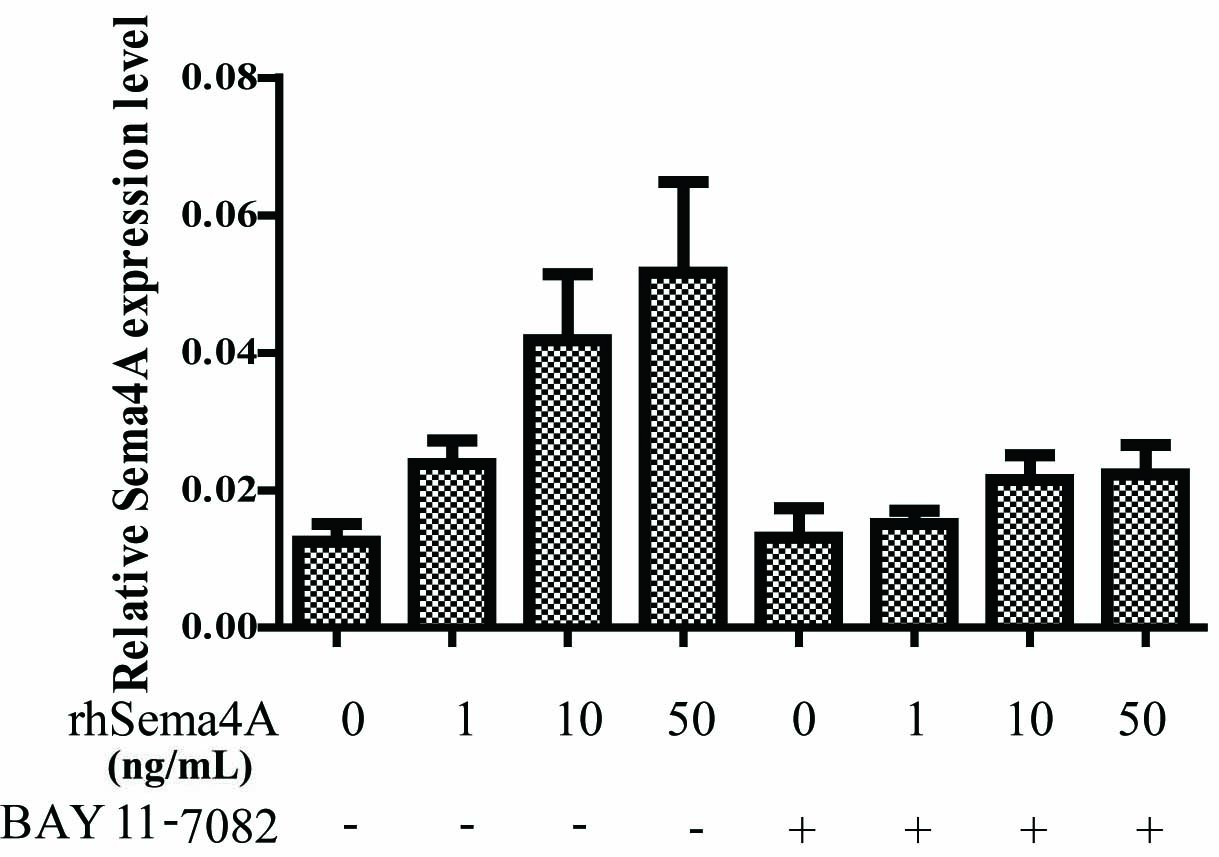

Supplement: Additional file 1: Figure S1. — Recombinant human semaphorin 4A (rhSema4A) treatment induced its own expression in synovial fibroblasts of rheumatoid arthritis (RASFs). (JPEG 759 kb) [file 13075_2015_734_MOESM1_ESM.jpeg]
